# Nanobodies Targeting the GP4 Protein Inhibit PRRSV Replication

**DOI:** 10.3390/microorganisms13112524

**Published:** 2025-11-02

**Authors:** Wenxiang Zhang, Aodi Wu, Honghuan Li, Tao He, Qianqian Dong, Hanwen Zhang, Jie Chen, Song Jiang, Jinliang Sheng

**Affiliations:** College of Animal Science and Technology, Shihezi University, Shihezi 832003, China; z302729814@163.com (W.Z.); 15739592133@163.com (A.W.); lhh121004@126.com (H.L.); ht960704@163.com (T.H.); 18699332880@163.com (Q.D.); aa747582693@163.com (H.Z.); 18381680727@163.com (J.C.)

**Keywords:** PRRSV, nanobody, GP4, antiviral activity

## Abstract

Porcine reproductive and respiratory syndrome virus (PRRSV) infection inflicts enormous economic losses on the global swine industry and imposes significant pressure on agricultural production. However, there are currently no clinically approved effective therapeutics specifically targeting PRRSV. Accordingly, the development of novel antiviral agents against PRRSV is urgently needed. Notably, the structural glycoprotein 4 (GP4) of PRRSV—which plays a crucial role in viral entry into host cells—represents a promising target for antiviral development. Nanobodies, characterized by their small size, structural stability, high affinity, and excellent solubility, have emerged as attractive candidates for next-generation therapeutic development. Yet, to date, no specific nanobodies targeting PRRSV GP4 have been reported. In this study, we isolated GP4-specific nanobodies using phage display technology and investigated their mechanisms underlying viral suppression through a series of in vitro functional assays. Our results demonstrate that Nb6, Nb31, and Nb85 significantly inhibit PRRSV infection by disrupting both viral attachment to host cells and subsequent internalization processes. Collectively, these findings indicate that Nb6, Nb31, and Nb85 hold substantial potential for development as antiviral agents against PRRSV infection.

## 1. Introduction

Porcine Reproductive and Respiratory Syndrome (PRRS) ranks among the most economically significant diseases impacting global swine production. It is characterized by reproductive failure in sows, respiratory distress across all production stages, and persistent infections in piglets, leading to substantial economic losses in major pig-producing countries worldwide [1,2,3]. The economic impact is profound, with annual losses estimated at approximately $664 million in the United States alone, while in Europe and Asia, the financial burden varies significantly depending on outbreak severity and production systems [4,5,6]. These losses stem from reduced reproductive performance, increased mortality rates in weaned pigs, and the costs associated with vaccination programs and biosecurity measures. The continuous emergence of novel recombinant and mutant viral strains, coupled with the limited efficacy of current vaccines and the absence of effective antiviral therapies [5,7,8,9,10], has complicated PRRS control efforts. Therefore, the development of effective therapeutic interventions or preventive measures is urgently needed to mitigate the impact of PRRSV on swine production.

PRRSV is broadly categorized into two major genotypes: PRRSV-1, which originated in Europe, and PRRSV-2, of North American origin. Both belong to the family *Arteriviridae* within the order *Nidovirales*. The viral genome consists of a single-stranded, positive-sense RNA molecule approximately 15.4 kb in length, containing at least 10 open reading frames (ORFs) that encode 8 structural proteins and 12 non-structural proteins (nsp1–nsp12) [11]. GP4 is one of four transmembrane glycoproteins encoded within the structural gene region. Notably, PRRSV utilizes glycoproteins, including GP4, to interact with the host receptor CD163, facilitating viral entry and infection [12].

GP4 is a key envelope glycoprotein of PRRSV and is essential for viral structure and infectivity [13,14]. Comprising 178–183 amino acid residues and containing four potential N-glycosylation sites, GP4 relies on these glycosylation modifications for full functionality, as their loss markedly impairs viral infectivity. GP4 assembles with GP2a, GP3, and GP5 into a heterotetrameric complex (GP2a-GP3-GP4-GP5) within the endoplasmic reticulum (ER). The subsequent GP4-dependent trafficking of this complex from the ER to the Golgi apparatus is a prerequisite for its integration into the viral envelope [15]; consequently, the absence of GP4 abrogates viral infectivity. Furthermore, GP4 colocalizes with the host receptor CD163 within lipid raft microdomains of the plasma membrane, which may promote efficient viral entry by concentrating receptor molecules [16]. The glycosylphosphatidylinositol (GPI) anchor modification of GP4 is essential for CD163 binding and viral entry, as mutations in key residues required for GPI anchoring (e.g., M162) abolish PRRSV infectivity [17]. Therefore, GP4 functions as a critical surface-exposed mediator of specific host receptor binding, viral adsorption, and penetration [18], establishing it as a prime target for anti-PRRSV therapeutic development.

Nanobodies (Nbs), or single-domain antibodies, consist solely of the heavy-chain variable domain (VHH) and were first discovered in camelids [19]. They exhibit advantageous properties such as small size, high specificity, thermal stability, excellent refolding capacity, and enhanced tissue penetration [20,21]. Furthermore, Nbs can be efficiently produced at high yields in both prokaryotic and eukaryotic expression systems [22,23]. Owing to their small molecular size and unique spatial conformation, Nbs are capable of recognizing and binding to cryptic epitopes or topological clefts on target proteins—regions that are typically inaccessible to conventional antibodies [24]. These properties make Nbs promising therapeutic candidates for a range of viral diseases. For instance, Nbs targeting viruses such as human immunodeficiency virus (HIV), severe acute respiratory syndrome coronavirus 2 (SARS-CoV-2), and respiratory syncytial virus (RSV) have been shown to significantly inhibit viral replication and proliferation in host cells, demonstrating potent antiviral activity [25,26,27]. In the context of PRRSV, recent studies have highlighted the efficacy of nanobodies targeting viral proteins such as the nucleocapsid (N) protein and non-structural proteins (e.g., nsp9), which can inhibit viral replication by blocking essential protein–protein interactions or viral assembly processes [28,29]. This underscores the potential of nanobodies as a novel antiviral strategy against PRRSV.

In this study, we identified five distinct Nb clones via alpaca immunization, construction of a phage display library, and solid-phase panning. These Nbs were recombinantly expressed in *Escherichia coli* (*E. coli*). Three of the soluble Nbs demonstrated potent neutralizing activity and inhibited PRRSV infection. Further mechanistic investigations revealed that Nb31 inhibits PRRSV infection specifically by targeting the viral adsorption and internalization processes. Collectively, our results identify Nb6, Nb31, and Nb85 as promising therapeutic candidates for combating PRRSV infections. The development of such nanobody-based therapeutics could provide a much-needed tool to reduce the economic burden of PRRS on the global swine industry, offering advantages in terms of production scalability, cost-effectiveness, and potential for rapid deployment against emerging viral strains [30,31]. Future applications may include not only therapeutic treatments but also diagnostic tools and prophylactic measures, ultimately contributing to more sustainable and resilient swine production systems.

## 2. Materials and Methods

### 2.1. Cells and Viruses

MARC-145 cells were purchased from Beina Chuanglian Biotechnology Co., Ltd. (BNCC, Xinyang, China) and cultured at 37 °C in a humidified atmosphere containing 5% CO_2_. The culture medium consisted of Dulbecco’s Modified Eagle Medium (Life Technologies Corporation, New York, NY, USA) supplemented with 10% fetal bovine serum (Gibco, Carlsbad, CA, USA) and 1% antibiotic–antimycotic solution (Solarbio, Beijing, China).

The PRRSV XJSW2021 strain (GenBank accession number: OR247780.1) was kindly provided by the research group of Professor Chuangfu Chen. Viral propagation was carried out in MARC-145 cells according to the following procedure: cells were seeded in T75 cell culture flasks and grown to approximately 80% confluency. The cells were then inoculated with PRRSV at a multiplicity of infection (MOI) of 0.1, diluted in DMEM, and incubated at 37 °C for 2 h. After incubation, the inoculum was removed and replaced with DMEM supplemented with 2% FBS. The infected cells were cultured for an additional 96 h until extensive cytopathic effects (CPEs) were observed. Culture medium and cells were harvested together and subjected to five freeze–thaw cycles. The resulting mixture was centrifuged at 8000 × *g* for 30 min at 4 °C to remove cellular debris. The supernatant was collected, filtered through a 0.22 μm membrane filter, and aliquoted into 1.5 mL microcentrifuge tubes. The viral titer was determined using the 50% tissue culture infective dose (TCID_50_) assay, and aliquots were stored at −80 °C for subsequent experiments.

### 2.2. Expression and Purification of PRRSV GP4 Recombinant Protein

A truncated sequence of GP4 with optimal properties was selected, and the corresponding plasmid (pSumo-mut-GP4) was synthesized by Zhongding Biotechnology Co., Ltd. (Nanjing, China). The pSumo-mut-GP4 plasmid was transformed into *E. coli* Transetta (DE3) competent cells (TransGen Biotech, Beijing, China). Transformed cells were cultured in Terrific Broth (TB medium: 12 g/L tryptone, 24 g/L yeast extract, 17 mM KH_2_PO_4_, 72 mM K_2_HPO_4_, and 0.4% sterile glycerol) until the optical density at 600 nm (OD_600_) reached 0.6. Protein expression was induced by adding isopropyl β-D-1-thiogalactopyranoside (IPTG) to a final concentration of 0.25 mM, followed by incubation at 16 °C for 16 h. Bacterial cells were harvested by centrifugation at 8000 × *g* at 4 °C for 10 min and resuspended in ultrasonic lysis buffer (50 mM NaH_2_PO_4_·2H_2_O, 300 mM NaCl, 10 mM imidazole, pH 8.0). After ultrasonication and subsequent centrifugation, the expression and solubility of the recombinant protein were analyzed by sodium dodecyl sulfate–polyacrylamide gel electrophoresis (SDS-PAGE). The supernatant was loaded onto a High Affinity Ni-NTA Resin column (TransGen Biotech, Beijing, China), and the target protein was eluted with 250 mM imidazole. The purified GP4-His recombinant protein was verified by SDS-PAGE and Western blotting. Additionally, Western blotting was performed to confirm the specific binding activity of the recombinant GP4 protein to PRRSV-positive serum.

### 2.3. Alpaca Immunization and Library Construction

Prior to immunization, pre-immune serum (2 mL) was collected from an adult male alpaca to serve as a negative control. For the primary immunization, 2 mg of recombinant GP4 protein was emulsified with an equal volume of Freund’s Complete Adjuvant (FCA) and administered via subcutaneous injection. Booster immunizations were administered every two weeks using 1 mg of antigen emulsified with Freund’s Incomplete Adjuvant (FIA), following the same emulsification procedure; this booster regimen was repeated four times [32]. Following the final immunization, serum was collected, and antibody titers were determined by indirect ELISA. Briefly, 96-well microplates were coated overnight at 4 °C with 4 μg/mL of recombinant GP4-His protein in carbonate-bicarbonate buffer (pH 9.6). Serial dilutions of alpaca serum were added as the primary antibody and incubated at 37 °C for 1.5 h. After washing with PBST, HRP-conjugated Goat Anti-Llama IgG H&L (Abcam, Cambridge, UK) was applied at a 1:50,000 dilution. Absorbance was measured at 450 nm, and the antibody titer was defined as the highest serum dilution that yielded an absorbance value twice that of the pre-immune serum, calculated using GraphPad Prism 10.1.2.

Three days after the fifth immunization, 100 mL of whole blood was collected from the alpaca, and peripheral blood mononuclear cells (PBMCs) were isolated using a Camelid Peripheral Blood Lymphocyte Separation Kit (TBD, Tianjin, China). Total RNA was extracted from PBMCs, and 5 μg of RNA was reverse-transcribed into cDNA using the HiFiScript SuperFast gDNA Removal cDNA Synthesis Kit (CWBIO, Beijing, China). The VHH gene was amplified via nested PCR: the first round used primers CALL001/CALL002 to generate a ~700 bp product, and the second round employed primers Cam-For-*sfi* I/Cam-Rev-*sfi* I to yield a ~400 bp fragment encoding the VHH domain [32]. The amplified product was ligated into the *Sfi* I-digested pComb3Xss phage display vector (AlpVHHs, Chengdu, China) and electrotransformed into *E. coli* TG1 electrocompetent cells. Transformed cells were collected, and 10 μL of the culture was serially diluted for titer determination; the remainder was supplemented with glycerol to a final concentration of 20% and stored at −80 °C. To calculate the library size, dilutions ranging from 10^−5^ to 10^−7^ were plated on ampicillin-containing LB agar. After incubation, colonies were counted, and the library capacity was determined using the formula: Library capacity = number of colonies × dilution factor × total volume (mL).

### 2.4. Screening of Specific Nanobodies

Phage display technology was employed to screen for PRRSV-GP4-specific nanobodies through four rounds of solid-phase panning, with modifications to a previously described protocol [32]. In brief, recombinant GP4 protein was diluted to 20 μg/mL in carbonate buffer, and the coating concentration was sequentially reduced across rounds: 20 μg/mL (Round 1), 10 μg/mL (Round 2), 5 μg/mL (Round 3), and 2.5 μg/mL (Round 4). A 100 μL aliquot of diluted antigen was added per well of a 96-well ELISA plate and coated overnight at 4 °C. The following day, wells were washed five times with PBST (PBS containing 0.05% Tween-20) and blocked with 200 μL of blocking agent, which varied by round: 3% bovine serum albumin (Solarbio, Beijing, China), 5% non-fat milk powder (Solarbio, Beijing, China), or serum-free blocking buffer (Servicebio, Wuhan, China). After blocking at 37 °C for 2 h, wells were washed again, and 100 μL of the phage-displayed VHH library (5.0 × 10^12^ pfu/mL) was added and incubated at 37 °C for 1 h. Unbound phages were removed by washing with PBST, and bound phages were eluted using 100 μL of 0.1 M triethylamine (Merck, Germany) per well for 10 min at room temperature. The eluate was immediately neutralized with an equal volume of 1 M Tris-HCl (pH 7.4), and phage titers were determined. Eluted phages were amplified for use in subsequent rounds, with a total of four pannings performed.

### 2.5. Nanobody Prokaryotic Expression

Five selected nanobody clones were expressed recombinantly in *E. coli*. Plasmids containing nanobody genes were extracted using the EasyPure^®^ HiPure Plasmid MiniPrep Kit (TransGen Biotech, Beijing, China) and transformed into TOP10F′ chemically competent cells (Weidi, Shanghai, China). Transformed cultures were grown in 2YT medium supplemented with ampicillin until OD_600_ reached 0.6. Nanobody expression was induced with 0.1 mM IPTG, followed by incubation at 37 °C with shaking at 220 rpm for 16 h. Cells were harvested by centrifugation at 7000× *g* for 10 min, lysed, and the supernatant was collected, filtered through a 0.45 μm membrane on ice, and subjected to purification using High Affinity Ni-NTA Resin (TransGen Biotech, Beijing, China) under the same conditions as those applied for recombinant GP4. The purified soluble Nbs-HA were analyzed by SDS-PAGE to confirm molecular weight, and Western blotting was performed using an anti-HA Tag Recombinant Antibody (Proteintech, Wuhan, China) to verify nanobody identity and binding activity.

### 2.6. Nanoantibody Affinity Testing

To evaluate the binding capacity of soluble nanobodies to recombinant GP4 protein, an indirect enzyme-linked immunosorbent assay (iELISA) was performed. Briefly, 96-well microplates were coated with recombinant GP4 antigen across a range of concentrations. A randomly selected soluble nanobody (20 μg/mL) was added to each well and incubated for 1.5 h. After five washes with PBST (PBS containing 0.05% Tween-20), an HA Tag Recombinant Antibody was applied as the secondary antibody at dilution ratios of 1:5000, 1:10,000, 1:15,000, and 1:20,000, followed by incubation for 1.5 h. For detection, horseradish peroxidase (HRP)-conjugated goat anti-rabbit IgG (Abmart, Shanghai, China) was used as the tertiary antibody at dilutions of 1:3000, 1:5000, and 1:8000, with incubation for 1.5 h. Tetramethylbenzidine (Sigma, St. Louis, MO, USA) was used as the chromogenic substrate, and the reaction was stopped with 3 M H2SO4 Absorbance was measured at 450 nm using a microplate reader (Thermo Fisher Scientific, Waltham, MA, USA). Checkerboard titration was first conducted to optimize the concentrations of coating antigen, secondary antibody, and tertiary antibody. Under these optimized conditions, the binding affinities of different soluble nanobodies were tested across a dilution series (10 μg/mL to 0.00001 μg/mL) using the same iELISA protocol.

### 2.7. Cytotoxicity Assay

The effect of soluble nanobodies on MARC-145 cell viability was assessed using a Cell Counting Kit-8 (Vazyme, Nanjing, China) with modifications to a reported method [33]. MARC-145 cells were seeded into 96-well plates at 1 × 10^4^ cells per well and cultured at 37 °C under 5% CO_2_ until a confluent monolayer formed. The spent medium was discarded, and cells were washed twice with PBS. Soluble nanobodies were diluted in serum-free DMEM to four concentrations (5, 10, 20, and 40 μM) and added to the wells, with six replicates per concentration. Untreated MARC-145 cells served as the normal control, and DMEM-only wells served as the blank control. After 48 h of incubation at 37 °C under 5% CO_2_, the medium was replaced with maintenance medium (DMEM with 2% FBS). Then, 10 μL of CCK-8 reagent was added to each well, followed by incubation for 1.5 h under the same conditions. Absorbance at 450 nm was measured to determine cell viability.

$$Cell\;viability(\%)=\frac{Nanoantibody\;group-Blank\;group}{Normal\;control-Blank\;group}\times 100\%$$

### 2.8. Quantitative Real-Time PCR

Total RNA was extracted from infected MARC-145 cells using TRIzol reagent (TransGen Biotech, Beijing, China) after three washes with PBS. Reverse transcription was carried out with the PrimeScript RT Master Mix kit (TaKaRa, Dalian, China) according to the manufacturer’s instructions. Quantitative real-time PCR (qPCR) was performed on a LightCycler^®^ instrument (Roche Diagnostics, Indianapolis, IN, USA) using ChamQ Universal SYBR qPCR Master Mix (Vazyme, Nanjing, China), nuclease-free water (TransGen Biotech, Beijing, China), and the ORF7-specific primer pair ORF7-F/ORF7-R [33]. Each 20 μL reaction was run in technical duplicates, with the β-actin gene serving as the endogenous control. Relative expression of viral ORF7 was calculated using the 2^−ΔΔCT^ method; all primer sequences used are provided in Table 1.

### 2.9. Virus Titration

Viral progeny production was quantified by end-point titration assay as previously described with modifications [33]. Briefly, MARC-145 cells were seeded in 96-well plates at 1 × 10^4^ cells per well and grown to confluence. Supernatants from infected cultures were serially diluted 10-fold in serum-free DMEM, and 100 μL of each dilution was applied to cell monolayers (*n* = 8 replicates per dilution). After 1.5 h of adsorption at 37 °C, the inoculum was replaced with maintenance medium (DMEM supplemented with 2% FBS). Cells were incubated for 5 days, and CPEs was recorded prior to calculation of the TCID_50_ by the Reed–Muench method.

### 2.10. Western Blotting Method

Following collection, cells were lysed using ice-cold RIPA buffer. A total of 25 μg of protein from each sample was separated by 12.5% SDS-PAGE and electrophoretically transferred to a polyvinylidene difluoride (PVDF) membrane (Millipore Sigma, Burlington, MA, USA). The membrane was blocked overnight at room temperature in Tris-buffered saline containing 0.01% Tween-20 (TBST) with 5% non-fat milk. Subsequently, the membrane was incubated for 2 h at room temperature with the following primary antibodies: rabbit anti-HA Tag recombinant antibody (1:15,000 dilution; ProteinTech, Wuhan, China), PRRSV N protein monoclonal antibody (1:2500 dilution; JNT, Beijing, China), and anti-β-actin antibody (1:5000 dilution; Cat. No. 66031-1; ProteinTech, Wuhan, China). After three washes with TBST, the membrane was incubated for 2 h at room temperature with appropriate horseradish peroxidase (HRP)-conjugated secondary antibodies, including goat anti-mouse IgG, goat anti-rabbit IgG, and goat anti-pig IgG (all at 1:5000 dilution; ProteinTech, Wuhan, China). Protein bands were visualized using an enhanced chemiluminescence (ECL) substrate (Vazyme, Nanjing, China), and chemiluminescent signals were captured using a ChemiDoc MP Imaging System (Bio-Rad, Hercules, CA, USA).

### 2.11. Neutralizing Effect of Nanobodies on Viruses

MARC-145 cells were seeded in 6-well plates at a density of 2 × 10^5^ cells per well and allowed to adhere overnight. PRRSV (MOI = 0.1) and nanobodies (10 μM) were pre-incubated together in serum-free DMEM at 37 °C for 1.5 h. The spent medium was then aspirated from the cells, and the pre-incubated PRRSV-nanobody mixture was added to the wells for a 1.5 h adsorption period at 37 °C. Following adsorption, the inoculum was removed and replaced with maintenance medium consisting of DMEM supplemented with 2% fetal bovine serum (FBS). To evaluate the time-dependent neutralization efficacy of the nanobodies, cells were harvested at 2, 4, 8, and 16 h post-infection (hpi) for subsequent analysis.

### 2.12. Inhibitory Effect of Nanobodies on Viruses

For subsequent analyses, the experimental procedures detailed in Section 2.11 were followed. Cell culture supernatants were collected at 24 and 48 hpi for quantification of progeny virus titers. Additionally, at 48 hpi, cells were harvested and subjected to Western blot analysis.

### 2.13. Determination of the Virus Life Cycle

For the viral attachment assay, MARC-145 cells were cultured in 6-well plates to a density of 2 × 10^5^ cells per well. Cells were pre-treated with 10 μM Nb31 for 1.5 h at 37 °C, followed by the addition of PRRSV (MOI = 0.1). Infection was carried out at 4 °C for 2 h to permit viral attachment while preventing internalization. Unbound virus was removed by washing three times with ice-cold PBS. Cells were then harvested and cell-associated viral RNA was quantified by PT-qPCR.

To evaluate viral internalization, MARC-145 cells were seeded in 6-well plates at 2 × 10^5^ cells per well and incubated with PRRSV (MOI = 0.1) for 1.5 h at 4 °C to synchronize attachment. After removal of unbound virus by washing with cold PBS, 10 μM Nb31 in DMEM was added and incubation continued at 37 °C for 2 h to allow internalization. To dissociate externally attached viruses, cells were treated with acidic PBS (pH 3.0), washed, collected, and subjected to PT-qPCR analysis for internalized viruses.

For the viral replication assay, MARC-145 cells (2 × 10^5^ cells per well in 6-well plates) were infected with PRRSV (MOI = 0.1) for 1.5 h at 37 °C. Following infection, non-internalized virus was inactivated by acidic PBS (pH 3.0) washing. Cells were then cultured in DMEM containing 10 μM Nb31 for 24 h at 37 °C before being harvested for quantification of viral replication via PT-qPCR.

Viral release was assessed by infecting MARC-145 cells (2 × 10^5^ cells per well) with PRRSV (MOI = 0.1) for 1.5 h at 37 °C. After washing with PBS to remove unabsorbed virus, cells were maintained in DMEM with 2% FBS for 24 h. The medium was then replaced with fresh medium containing 10 μM Nb31 and incubated for another 2 h. Supernatants were collected and viral titers were determined by TCID_50_ assay [34].

### 2.14. Molecular Docking

The tertiary structures of GP4 and the nanobodies (Nb6, Nb31, and Nb85) were predicted using the Robetta server “http://robetta.bakerlab.org/ (accessed on 30 June 2025)”, an established platform for protein structure prediction [35]. Protein–protein interaction interfaces between GP4 and each nanobody were subsequently analyzed using PDBe PISA “https://www.ebi.ac.uk/pdbe/pisa/ (accessed on 30 June 2025)”, a web-based tool for probing macromolecular interfaces [36]. Structural models of the resulting complexes were visualized using PyMOL 2.6.0a0 (Schrödinger, LLC, New York, NY, USA), a widely used molecular graphics system.

### 2.15. Statistical Analysis

All data are representative of at least three independent biological replicates. Statistical analyses were performed using GraphPad Prism version 10.1.2 (GraphPad Software, San Diego, CA, USA). Depending on experimental design, one-way ANOVA, two-way ANOVA, or unpaired Student’s *t*-test were applied as appropriate. Data are presented as mean ± standard deviation (SD). A *p*-value less than 0.05 was considered statistically significant, with notations as follows: ns, not significant (*p* > 0.05); * *p* ≤ 0.05; ** *p* ≤ 0.01; *** *p* ≤ 0.001. Densitometric analysis of Western blot bands was conducted using ImageJ 1.53k software (National Institutes of Health, Bethesda, MD, USA).

## 3. Results

### 3.1. Preparation of GP4 Antigen Protein and WB Validation

**Figure 1:**
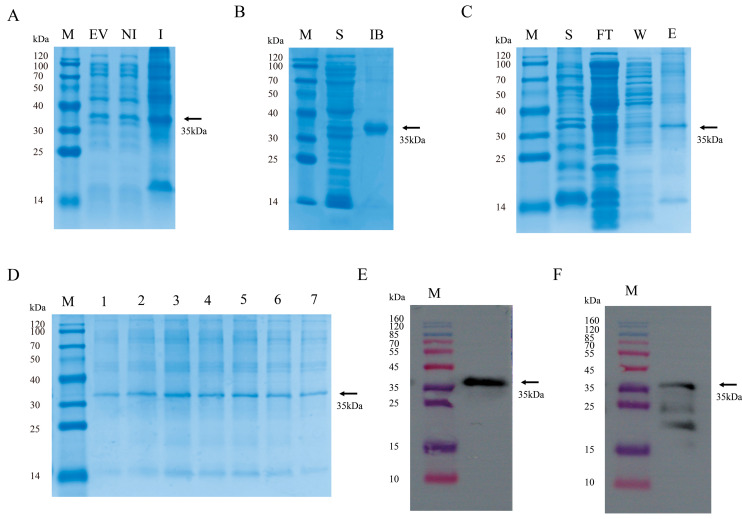
Expression, purification, and characterization of the PRRSV GP4 recombinant protein. (**A**) SDS-PAGE analysis of recombinant protein expression. Lane M: Protein marker; Lane EV: pSumo-mut-GP4 vector control; Lane NI: pSumo-mut-GP4 before induction; Lane I: pSumo-mut-GP4 after induction. (**B**) SDS-PAGE analysis of recombinant protein expression localization. Lane S: Supernatant from cell lysate; Lane IB: Precipitate from cell lysate. (**C**) SDS-PAGE purification of recombinant proteins. Lane FT: permeate; Lane W: wash buffer; Lane E: elution buffer. (**D**) SDS-PAGE elution peaks of recombinant proteins. Lanes 1–7: recombinant proteins collected in different Eppendorf tube. (**E**) Reaction of recombinant proteins with His antibody. (**F**) Reaction of recombinant proteins with PRRSV-positive serum.

Bacteria were cultured in TB medium until the OD_600_ reached 0.6, after which expression of the pSumo-mut-GP4 protein was induced by adding 0.25 mM IPTG and incubating at 16 °C. The soluble fraction of the cell lysate was purified using High Affinity Ni-NTA Resin. SDS-PAGE analysis indicated that the molecular weight of the purified GP4-His recombinant protein was approximately 35 kDa (Figure 1A–D). Western blotting further confirmed the expression and purification of the recombinant protein using both an anti-His monoclonal antibody and PRRSV-positive serum, each revealing a specific band around 35 kDa (Figure 1E,F).

### 3.2. VHH Library Construction

**Figure 2:**
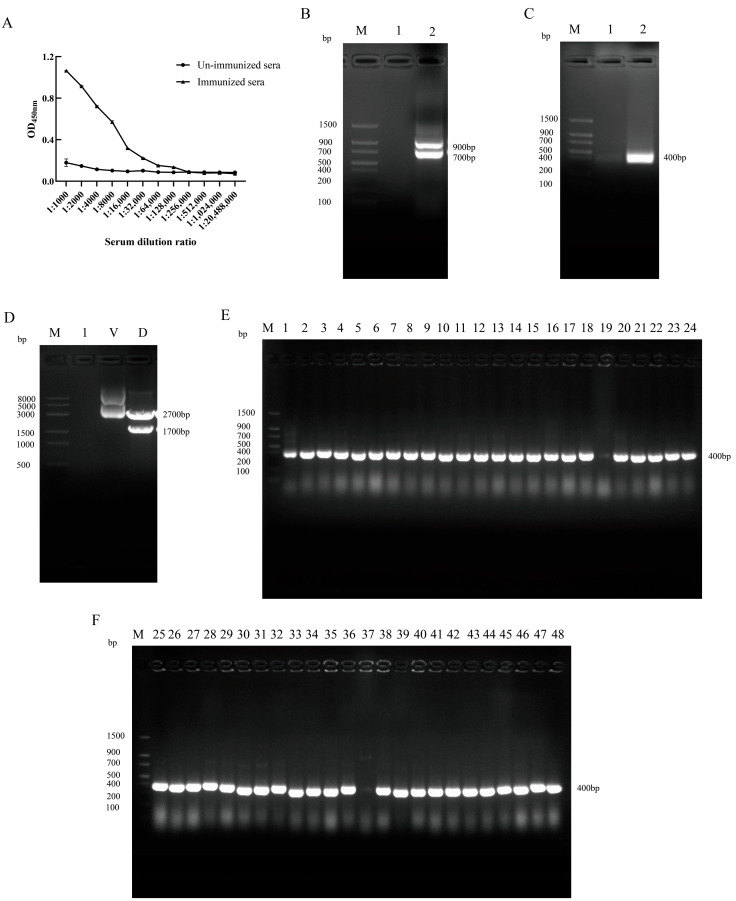
Serum titer determination, VHH library construction, and positive rate identification. (**A**) iELISA: Serum antibody titers before and after immunization. Data are presented as the mean ± SD of three independent experiments conducted in triplicate. (**B**) Nest PCR single-round amplification results. Lane 1: Blank control; Lane 2: Amplification products of 900 bp and 700 bp. (**C**) Nest PCR second-round amplification results. Lane 1: Blank control; Lane 2: Amplification products of 400 bp. (**D**) pComb3Xss double digestion results. Lane 1: Blank control; Lane V: Undigested plasmid; Lane D: double-digested plasmid, approximately 1700 and 2700 bp in size. (**E**) Bacterial culture PCR positivity rate identification. Lanes 1−24: 24 different monoclonal antibodies, lane 19 was negative. (**F**) Bacterial culture PCR positivity rate identification. Lanes 25−48: 24 different monoclonal antibodies, lane 37 was negative.

The purified GP4-His recombinant protein was used both for camelid immunization and as a coating antigen during bioassay selection. Serum samples collected before and after immunization were analyzed by iELISA to detect GP4-specific antibodies. The results indicated a post-immunization antibody titer of 1:128,000, with no specific reactivity observed in the pre-immunization serum (Figure 2A). A target DNA fragment of approximately 400 bp was amplified after two rounds of nested PCR. The VHH library was constructed by *Sfi* I double digestion and ligation, yielding a library with a capacity of approximately 8.4 × 10^12^ PFU/mL (Figure S1 and Figure 2B–D). Colony PCR analysis showed that 96% of the clones contained an insert of the expected size for the VHH gene (Figure 2E,F).

### 3.3. Separation and Identification of Nanobodies

**Figure 3:**
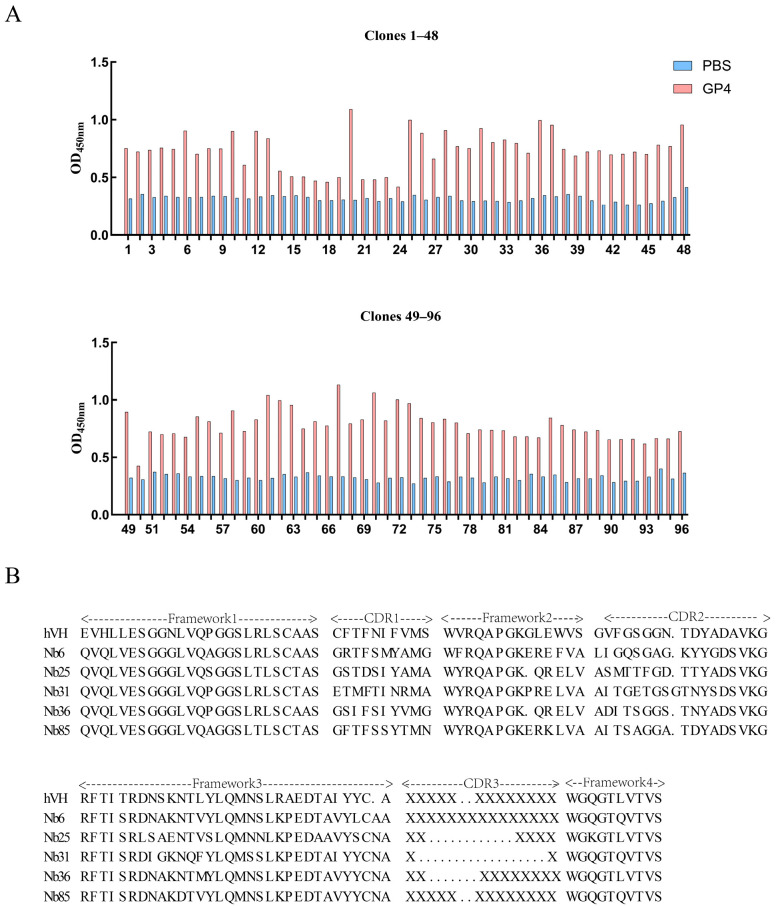
iELISA and nanobody sequencing results. (**A**) iELISA detection of GP4 recombinant protein specifically binding to nanobody crude extract. The cut-off value for GP4 relative to PBS is greater than 2. (**B**) Amino acid sequence alignment of GP4-specific nanobodies and human VH.

Solid-phase biopanning against recombinant GP4-His protein was conducted over four rounds, resulting in significant enrichment of VHH-displaying phage particles (Table 2). Ninety-six monoclonal clones isolated from the fourth round were screened by iELISA using crude nanobody extracts. A cut-off value of greater than 2 for GP4 relative to PBS was employed, with 85 out of 96 monoclonal antibodies exhibiting antigen-specific binding activity (Figure 3A). All positive clones were subjected to sequencing, which identified five unique nanobody sequences, designated Nb6, Nb25, Nb31, Nb36, and Nb85. These sequences were aligned and compared with human VHH framework regions to assess structural homology (Figure 3B).

### 3.4. Preparation of Soluble Nanobodies and Affinity Validation

**Figure 4:**
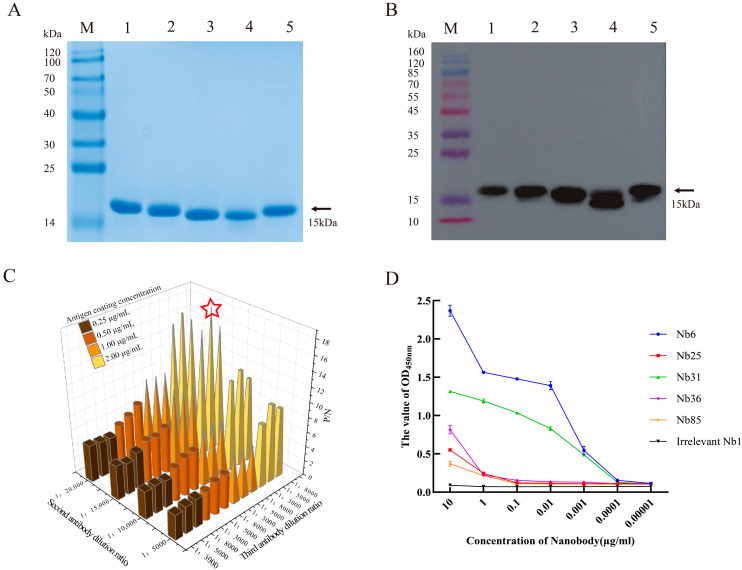
Expression, identification, and affinity testing of soluble nanobodies. (**A**) SDS-PAGE analysis of soluble nanobody expression. Lane M: protein marker; Lane 1: Nb6; Lane 2: Nb25; Lane 3: Nb31; Lane 4: Nb36; Lane 5: Nb85. (**B**) Reaction of soluble nanobodies with HA antibody. Lane order is consistent with SDS-PAGE. (**C**) Checkered titration assay, P/N: Optical density value of the test sample/Average optical density value of the negative control. (**D**) Titration of the binding affinity of five soluble nanobodies with GP4 recombinant protein at different dilution gradients using iELISA, with unrelated Nb1 as the negative control. Data are presented as the average ± SD of three independent experiments conducted in triplicate.

Recombinant plasmids pComb3Xss-Nbs-HA encoding five distinct nanobodies were transformed into TOP10F′ chemically competent cells for soluble expression. The transformed cells were cultured in 2YT/Amp medium until OD_600_ reached 0.6, followed by induction with 0.1 mM IPTG at 37 °C for 16 h. Cells were harvested by centrifugation, lysed, and the supernatant was collected for nanobody purification using the same protocol as applied for the GP4-His recombinant protein. All five nanobodies were successfully expressed in the soluble fraction, as confirmed by SDS-PAGE (Figure S2 and Figure 4A). Western blot analysis using an anti-HA monoclonal antibody detected a specific band of approximately 15 kDa, verifying the expression and purification of each nanobody (Figure 4B). Checkerboard titration was used to optimize assay conditions, identifying the following parameters: antigen coating concentration of 2 μg/mL, secondary antibody dilution of 1:15,000, and tertiary antibody dilution of 1:5000 (Figure 4C). The binding affinity of each nanobody to recombinant GP4-His was evaluated by iELISA. Nb6 and Nb31 exhibited high affinity, with detectable binding even at 0.1 μg/mL, whereas Nb25, Nb36, and Nb85 showed lower affinity, with binding observed only at 1 μg/mL. A negative control nanobody, Nb1, was included and showed no binding activity (Figure 4D).

### 3.5. Cytotoxicity Testing and Neutralization Effect of Nanobodies on Viruses

**Figure 5:**
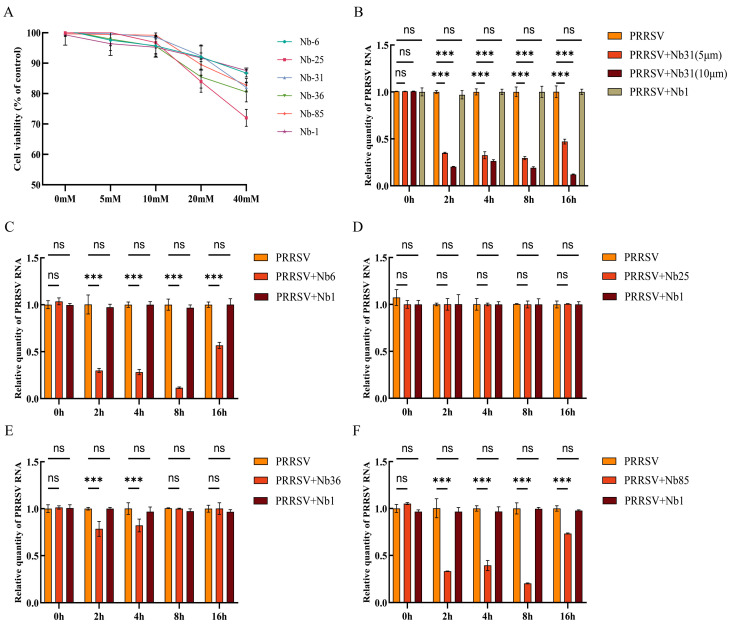
Cytotoxicity and neutralization effects. (**A**) Cytotoxicity of Nb6, Nb25, Nb31, Nb36, Nb85, and unrelated Nb1 on MARC-145 cells. (**B**) Neutralization activity of 5 μM and 10 μM Nb31 against the virus at 2, 4, 8, and 16 hpi. (**C**) Neutralization activity of 10 μM Nb6 against the virus at 2, 4, 8, and 16 hpi. (**D**) The neutralizing effect of 10 μM Nb25 on the virus at 2, 4, 8, and 16 hpi. (**E**) The neutralizing effect of 10 μM Nb36 on the virus at 2, 4, 8, and 16 hpi. (**F**) The neutralizing effect of 10 μM Nb85 on the virus at 2, 4, 8, and 16 hpi. The statistical method employed was one-way analysis of variance, data are presented as the average ± SD of three independent experiments conducted in triplicate. ns, not significant (*p* > 0.05); *** *p* ≤ 0.001.

The cytotoxicity of five soluble nanobodies toward MARC-145 cells was evaluated using the CCK-8 assay. After 48 h of co-incubation, cell viability remained comparable to that of untreated controls at nanobody concentrations up to 10 μM. However, a decline in overall viability was observed when the concentration was increased to 20 μM (Figure 5A), indicating that the nanobodies are non-cytotoxic at concentrations ≤10 μM within this timeframe. To assess antiviral activity, cells were treated with 5 μM or 10 μM of Nb31 and harvested at various time points for quantitative RT-PCR analysis. The results demonstrated a stronger neutralizing effect at 10 μM compared to 5 μM (Figure 5B). Among the five nanobodies tested at 10 μM, Nb6, Nb31, and Nb85 significantly reduced viral RNA levels as early as 2 hpi, indicating potent neutralization (Figure 5C,E,F). In contrast, Nb25 and Nb36 showed no significant antiviral effects at any time point (Figure 5D,F).

### 3.6. Detection of Viral Titer in Offspring

**Figure 6:**
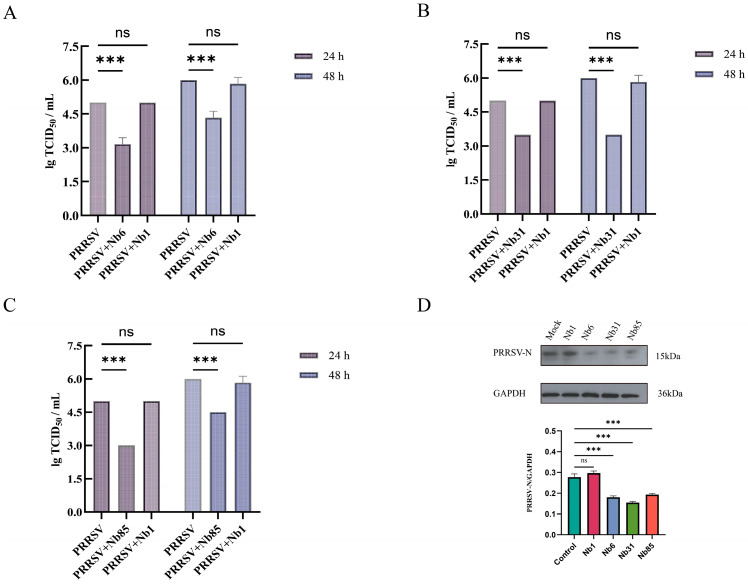
Inhibitory effects of Nb6, Nb31, and Nb85 on progeny viruses. (**A**) MARC-145 cells infected with Nb6 at 10 μM were treated for 24 h and 48 h, and the supernatant was collected to determine TCID_50_. (**B**) 10 μM Nb31 was added to infected MARC-145 cells at 24 h and 48 h post-infection, and the supernatant was collected to measure TCID_50_. (**C**) 10 μM Nb85 was added to infected MARC-145 cells at 24 h and 48 h post-infection, and the supernatant was collected to measure TCID_50_. (**D**) Intracellular viral load at 48 hpi was detected by Western blotting using an anti-N protein monoclonal antibody. The statistical method employed was one-way analysis of variance, data are presented as the average ± SD of three independent experiments conducted in triplicate. ns, not significant (*p* > 0.05); *** *p* ≤ 0.001.

To further evaluate the effect of intracellular nanobodies on PRRSV proliferation in MARC-145 cells, cells were infected with PRRSV at an MOI of 0.1. Supernatants were collected at 24 and 48 hpi, and progeny virus titers were quantified. Compared to the negative control nanobody Nb1, treatment with Nb6, Nb31, and Nb85 resulted in inhibition rates of 36.7%, 30.0%, and 30.0%, respectively, at 24 hpi. By 48 hpi, the inhibition rates for these nanobodies were 25.7%, 42.3%, and 22.9%, respectively. No significant difference in viral titer was observed between the Nb1-treated group and the untreated cell control (Figure 6A–C). Western blot analysis of cells harvested at 48 hpi showed a marked reduction in PRRSV N protein expression in groups treated with Nb6, Nb31, or Nb85 compared to both control groups (Figure 6D).

### 3.7. The Effect of GP4-Nb31 on the Viral Life Cycle

**Figure 7:**
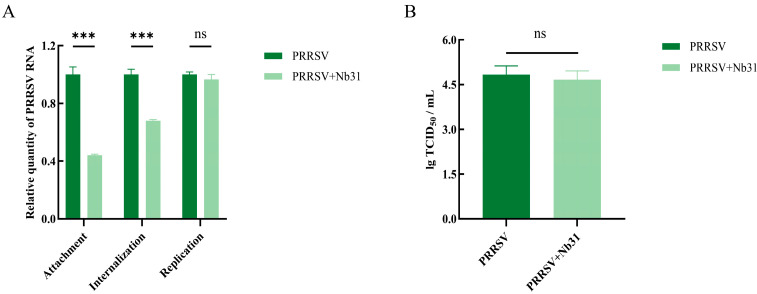
Investigation of the effect of Nb31 on the viral life cycle. (**A**) RT-qPCR analysis of PRRSV mRNA expression levels after treatment of MARC-145 with 10 μM Nb31, including attachment, internalization, and replication. (**B**) Analysis of the effect of 10 μM Nb31 treatment on PRRSV release after treatment of MARC-145 using TCID_50_. Data are presented as the average ± SD of three independent experiments conducted in triplicate. ns, not significant (*p* > 0.05); *** *p* ≤ 0.001.

To further elucidate the mechanism of Nb31-mediated inhibition of PRRSV, we systematically evaluated its impact on four distinct stages of the viral life cycle: attachment, internalization, replication, and release. Quantitative RT-PCR analysis demonstrated that Nb31 treatment significantly reduced PRRSV mRNA levels during both the viral attachment and internalization phases compared to the PRRSV-infected control group, whereas no significant inhibitory effect was observed during the replication phase (Figure 7A). Consistent with this, viral titers measured by TCID_50_ assay indicated that Nb31 did not significantly impair viral release (Figure 7B). These results collectively indicate that Nb31 inhibits PRRSV infection primarily by interfering with early stages of the viral life cycle, specifically attachment and internalization.

### 3.8. Molecular Docking of VHH and GP4 Recombinant Proteins

**Figure 8:**
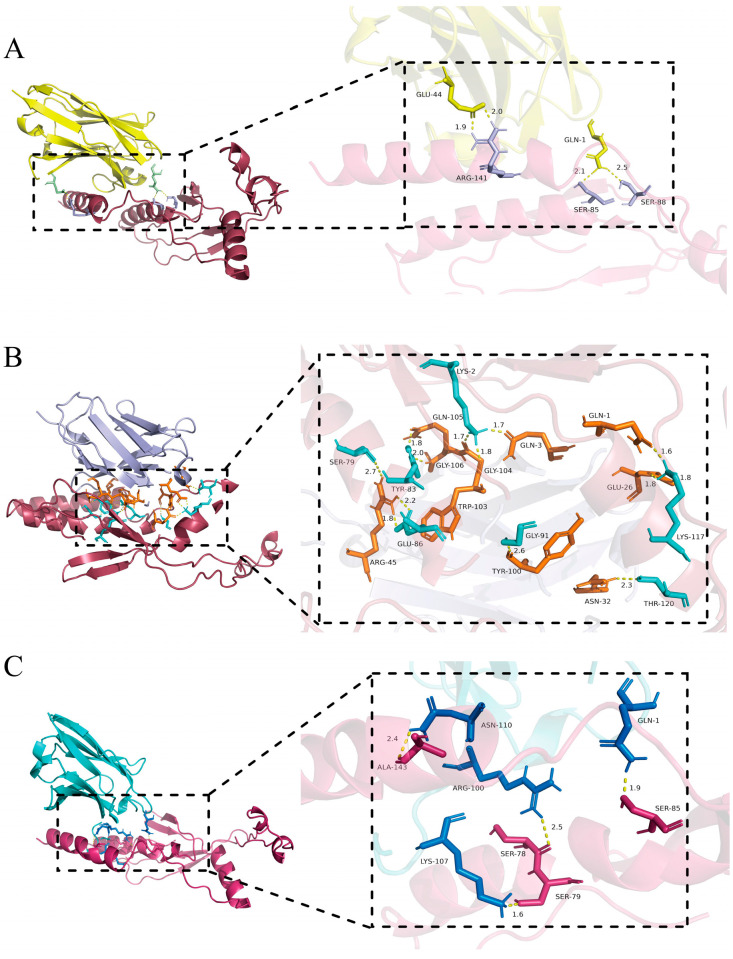
Molecular docking of nanobodies with antigen proteins. (**A**) GP4 protein docking results with Nb6 molecule (GP4: red; Nb6: yellow). (**B**) GP4 protein docking results with Nb31 molecule (GP4: red; Nb31: purple). (**C**) GP4 protein docking results with Nb85 molecule (GP4: red; Nb85: green).

Analysis using the PDBe PISA server revealed that the GP4–Nb6 complex is stabilized by four hydrogen bonds and four salt bridges, with a binding free energy (△G) of −12.6 kcal/mol (a △G value below −4.25 kcal/mol indicates significant binding activity between ligand and receptor) (Figure 8A, Tables S1 and S2). The GP4–Nb31 interaction involves 12 hydrogen bonds and five salt bridges, with a △G of −12.8 kcal/mol (Figure 8B, Tables S3 and S4), while GP4 and Nb85 are connected through five hydrogen bonds and exhibit a △G of −16.5 kcal/mol (Figure 8C, Table S5). Collectively, these molecular simulations confirm the high-affinity binding between GP4 and each of the three nanobodies (Nb6, Nb31, and Nb85), providing structural insights into their mechanisms as antiviral agents against PRRSV.

## 4. Discussion

Since its initial identification in the late 1980s, PRRSV has continued to impose substantial economic losses on the global swine industry [37,38]. In the absence of effective commercial vaccines that confer broad protection and the lack of specific antiviral therapies, the development of novel countermeasures remains urgent. Here, we report the development of nanobodies targeting the GP4 protein of PRRSV and demonstrate their potent inhibition of viral infection through specific blockage of viral adsorption and internalization.

Beyond their therapeutic potential demonstrated in this study, nanobodies offer promising perspectives for diverse applications. Their exceptional stability and modifiability make them ideal candidates for diagnostic development, including rapid field tests and imaging probes [39,40]. Furthermore, the possibility of engineering multi-specific nanobodies could address PRRSV’s high genetic variability by simultaneously targeting multiple viral epitopes or host factors [41,42,43]. The fusion of nanobodies with effector domains also presents opportunities for developing novel antiviral strategies that not only block viral entry but also trigger immune-mediated clearance of infected cells [41].

The affinity results may not exhibit a positive correlation with the viral inhibitory effect. The prokaryotically expressed Nb85 protein displayed low affinity for the recombinant GP4 protein but exhibited strong inhibitory effects in PRRSV neutralization assays. Conversely, Nb25 showed high affinity for the recombinant protein but demonstrated limited inhibitory effects against PRRSV. This discrepancy may arise because nanobodies typically target conformational or functional epitopes on viral proteins. However, the recombinant protein may lack the post-translational modifications or dynamic conformational changes present in the viral environment, preventing it from fully mimicking the natural three-dimensional conformation of the GP4 protein on the viral surface. Nb85 may bind a “conformational epitope” that is structurally incomplete in the recombinant protein but exposed on the intact virus, whereas Nb25 may target a “linear epitope” that is exposed on the recombinant protein but masked or glycosylated on the viral surface. This difference ultimately enables Nb85 to neutralize the virus [44,45,46]. Moreover, the glycosylation patterns of eukaryotic cells (such as virus-infected cells) and prokaryotic cells (bacteria expressing recombinant proteins) are completely different. Recombinant GP4 may lack critical glycans, directly compromising its binding to nanobodies. This may explain why Nb25 exhibits high affinity for recombinant proteins yet fails to neutralize the virus. In addition to competing with the native viral conformation, some nanobodies neutralize by agglutinating viral particles or inducing conformational changes [47,48]; however, such functions are not detectable in conventional affinity assays (e.g., ELISA). Therefore, in recombinant protein–based antibody screening, candidates showing low or no initial affinity should not be prematurely discarded. Instead, these candidates should undergo preliminary functional validation through live-virus neutralization assays to avoid overlooking potential therapeutic agents with unique mechanisms of action.

Nanobodies at 10 μM exhibited stronger viral neutralization than at 5 μM, confirming that their antiviral activity is dose dependent. A similar phenomenon was observed in the previously reported Nb6, which targets PRRSV Nsp9 [13]. Up to 16 h post infection, the nanobodies maintained significant neutralizing activity; however, by 48 h, the inhibitory effects of Nb6 and Nb85 declined, likely due to the continual accumulation of progeny viruses. Further mechanistic investigation revealed that Nb31 inhibits PRRSV primarily by blocking viral adsorption and internalization. GP4, which mediates PRRSV entry into host cells, interacts specifically with the host receptor CD163 to facilitate viral attachment and internalization [49]. Moreover, the GP4 complex can trigger viral endocytosis through CD163-mediated signaling—a process abolished in CD163-knockout cells [47,48]. These findings suggest that Nb31 may inhibit viral entry by interfering with GP4–CD163 binding or disrupting subsequent endocytotic signaling pathways.

Molecular docking analysis revealed that the receptor-binding domain (RBD) of GP4 engages the host receptor CD163 through salt-bridge-rich regions containing clusters of charged residues. Nb31 was found to form 12 hydrogen bonds and five salt bridges with GP4—the highest number of polar interactions among the tested nanobodies—indicating high-affinity and specific binding. Critically, the Nb31–GP4 binding interface shows substantial overlap with the predicted GP4–CD163 interaction domain, suggesting that Nb31 may competitively inhibit viral adsorption by sterically hindering GP4–CD163 complex formation [50]. Furthermore, the high-affinity engagement of Nb31 may induce a conformational lock in GP4, impairing its membrane fusion dynamics—a mechanism analogous to HIV gp41 fusion inhibitors that prevent six-helix bundle formation by binding the HR1 domain [44]. In contrast to nanobodies targeting internal viral proteins such as the nucleocapsid (e.g., N protein), which act during replication [28], the GP4-specific nanobody described here intercepts viral invasion at the earliest stage. This “front-line” neutralization strategy effectively reduces initial infection, limits intracellular viral load, suppresses subsequent transmission, and may mitigate the risk of viral escape.

While this study demonstrates the promising antiviral potential of GP4-targeted nanobodies in vitro, several limitations should be noted. The evaluation was conducted primarily in the MARC-145 cell line—a monkey kidney-derived model widely used in PRRSV research but distinct from native porcine alveolar macrophages (PAMs) in terms of receptor expression and immune functionality. This difference may affect the physiological relevance of the neutralization efficacy observed. Furthermore, all experiments were performed using a single PRRSV strain (XJSW2021, genotype 2), without inclusion of other epidemic strains such as NADC30-like or highly pathogenic PRRSV variants, nor representatives of PRRSV-1. Consequently, the breadth of neutralization across diverse viral genotypes remains unassessed. These limitations underscore the necessity for future studies to employ primary PAMs and a broader panel of PRRSV strains to more comprehensively evaluate the therapeutic potential of these nanobodies.

## 5. Conclusions

This study reports the first successful isolation of nanobodies targeting the GP4 protein of PRRSV from a VHH library. Systematic functional analyses demonstrated that these nanobodies effectively inhibit two critical stages of the PRRSV life cycle—viral adsorption and internalization—thereby significantly reducing infection efficiency. Three candidates, Nb6, Nb31, and Nb85, exhibited particularly strong antiviral activity, highlighting their potential as therapeutic agents against PRRSV. Furthermore, this work provides novel mechanistic insights into arterivirus neutralization strategies and lays a foundation for the development of targeted biologics against this important family of viral pathogens.

## 6. Patents

Patent applications for nanobodies are currently being filed.

## Figures and Tables

**Table 1:** Primers used in this study.

Primer Name	Sequence (5′–3′)	Purpose
GP4-F	CGGGATCCTGCAAACCATGTTTCTCCAGCT	pSumo-mut -GP4
GP4-R	AACTGCAGGATAGCCAGCAGAATCGCAAC	
CALL001	GTCCTGGCTGCTCTTCTACAAGG	
CALL002	GGTACGTGCTGTTGAACTGTTCC	
Cam-For-sfi I	CATGCCATGACTGTGGCCCAGGCGGCCCAGGTGCAGCTCGTGGAGTCTGGRGGAGG	^1^
Cam-Rev-sfi I	CATGCCATGACTCGCGGCCGGCCTGGCCGGAGACGGTGACCWGGGT	^1^
Pcomb3xss-F	AAGACAGCTATCGCGATTGCA G	Pcomb3xss
Pcomb3xss-R	GCCCCCTTATTAGCGTTTGCCATC	
ORF7-F	AGATCATCATCGCCCAACAAAAC	RT-qPCR
ORF7-R	GACACAATTGCCGCTCACTA	
β-actin-F	TCCCTGGAGAAGAGCTACGA	RT-qPCR
β-actin-R	AGCACTGTGTTGGCGTACAG	

^1^ Restriction sites are underlined.

**Table 2:** Enrichment of phage particles carrying GP4-specific Nbs.

Round of Panning	Input Phage (pfu/mL)	P Output (pfu/mL)	N Output (pfu/mL)	Recovery (P/Input)	P/N
1st round	5.00 × 10^12^	1.09 × 10^6^	2.00 × 10^4^	2.18 × 10^−7^	5.45 × 10^1^
2nd round	5.00 × 10^12^	3.13 × 10^7^	1.98 × 10^5^	6.26 × 10^−6^	1.58 × 10^2^
3rd round	2.12 × 10^12^	3.46 × 10^8^	2.41 × 10^6^	1.63 × 10^−4^	1.43 × 10^2^
4th round	5.00 × 10^12^	7.50 × 10^8^	1.05 × 10^6^	1.50 × 10^−4^	7.14 × 10^2^

## Data Availability

The data presented in this study are available in the article and Supplementary Materials.

## Supplementary Materials

The following supporting information can be downloaded at: https://www.mdpi.com/article/10.3390/microorganisms13112524/s1, Figure S1: Nanobody library capacity determination; Figure S2: Expression and purification of soluble nanobodies; Table S1: Hydrogen bonds between GP4 and Nb6; Table S2: Salt bridges between GP4 and Nb6; Table S3: Hydrogen bonds between GP4 and Nb31; Table S4: Salt bridges between GP4 and Nb31; Table S5: Hydrogen bonds between GP4 and Nb85.

## Abbreviations

The following abbreviations are used in this manuscript:

PRRSV	Porcine reproductive and respiratory syndrome virus
Nbs	Nanobodies
VHH	The heavy-chain variable domain
IPTG	Isopropyl β-D-1-thiogalactopyranoside
FCA	Freund’s Complete Adjuvant
FIA	Freund’s Incomplete Adjuvant
CPEs	cytopathic effects
TCID_50_	The 50% tissue culture infective dose
SDS-PAGE	sodium dodecyl sulfate–polyacrylamide gel electrophoresis
PBMCs	peripheral blood mononuclear cells
iELISA	indirect enzyme-linked immunosorbent assay
CDR	Complementarity-Determining Region
