# Supplementary material for: Nanobodies Targeting the GP4 Protein Inhibit PRRSV Replication
Source: Microorganisms. 2025 Nov 2;13(11):2524. doi: 10.3390/microorganisms13112524 (PMC12654830; doi:10.3390/microorganisms13112524)
Supplement: Supplementary file 1 [file microorganisms-13-02524-s001.zip › Ethical Certification.pdf]

石河子大学生物伦理委员会伦理审查同意函
Approval Letter of Biology Ethics Committee of Shihezi University

<table>
<tr><td>批件号
Approval Number</td><td colspan="4">A2024-721</td></tr>
<tr><td>项目名称
Project name</td><td colspan="4">TLR7/8 在绵羊肺腺瘤病理形成中的调节作用分子机制研究
(Molecular Mechanisms of TLR7/8 Regulation in the Pathological Formation of Sheep Pulmonary Adenomatosis)</td></tr>
<tr><td>项目来源
Sponsor</td><td colspan="4">国家自然基金（32460872）</td></tr>
<tr><td>研究单位
Research department</td><td colspan="4">石河子大学</td></tr>
<tr><td>主要研究者
Main researchers</td><td colspan="4">张文祥</td></tr>
<tr><td>审批材料清单
List of Documents</td><td colspan="4"></td></tr>
<tr><td rowspan="3">拟用动物情况
Decodes of the animals</td><td>动物类别/品系
Species of strain</td><td>等级
Grade</td><td>使用数量（♂/♀）
Number</td><td>动物来源
Animal sources</td></tr>
<tr><td>羊驼</td><td>普通</td><td>1（♀）</td><td>石河子大学动物科技学院</td></tr>
<tr><td></td><td></td><td></td><td></td></tr>
<tr><td>实验要点
Outline of experiments</td><td colspan="4">研究过程中将利用羊驼开展相关动物实验。利用实验动物免疫重组蛋白，提取血液中的淋巴细胞，用于构建纳米抗体噬菌体文库。本项目所有实验动物操作严格遵守《实验动物福利与伦理审查指南》、《实验动物饲养管理和使用指南》，并按照石河子大学实验动物伦理委员会制定的规章执行。所有实验动物使用经石河子大学实验动物伦理委员会批准。</td></tr>
<tr><td>审查意见
Evaluation Comments</td><td colspan="4">Approval 同 意</td></tr>
<tr><td>主任委员签字
Signature of Chairman</td><td colspan="4">张辉之印</td></tr>
<tr><td>伦理委员会
Ethics Committee</td><td colspan="4">石河子大学生物伦理委员会（盖章）</td></tr>
<tr><td>日期
Date</td><td colspan="4">2024 年 9 月 22 日</td></tr>
</table>
