# Supplementary material for: Nanobodies Targeting the GP4 Protein Inhibit PRRSV Replication
Source: Microorganisms. 2025 Nov 2;13(11):2524. doi: 10.3390/microorganisms13112524 (PMC12654830; doi:10.3390/microorganisms13112524)
Supplement: Supplementary file 1 [file microorganisms-13-02524-s001.zip › Supplementary material.pdf]

Nanobodies targeting the GP4 protein inhibit PRRSV replication

Wenxiang Zhang[1], Aodi Wu[1], Honghuan Li[1], Tao He[1], Qianqian Dong[1], Hanwen Zhang[1], Jie Chen[1], Song Jiang[1,*], Jinliang Sheng[1,*]

1 College of Animal Science and Technology, Shihezi University, 832003, China. z302729814@163.com (WZ); 1353499553@qq.com (AW); lhh121004@126.com (HL); ht960704@163.com (TH); 1696045598@qq.com (QD); aa747582693@163.com (HZ); jiachi0201@foxmail.com (JC)

* Correspondence: jsshzu@126.com (SJ); 1572621211@qq.com (JS)

Supplementary materials

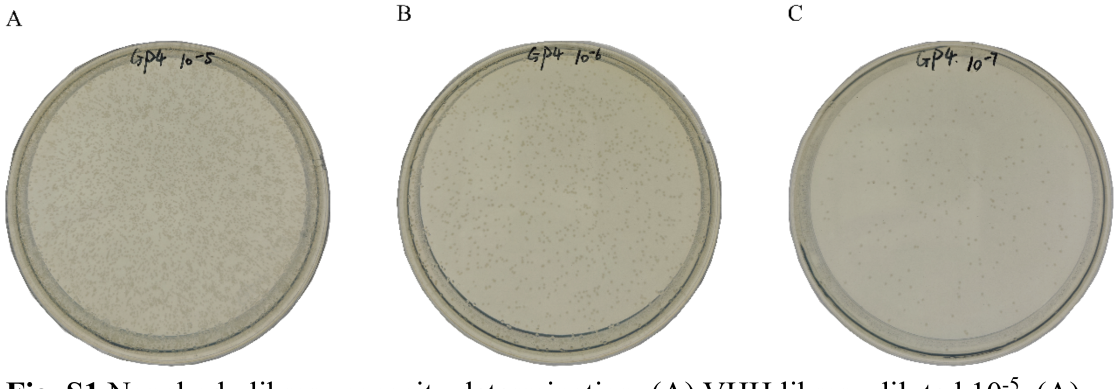

Fig. S1 Nanobody library capacity determination. (A) VHH library diluted 10^{-5}. (A) VHH library diluted 10^{-6}. (A) VHH library diluted 10^{-7}.

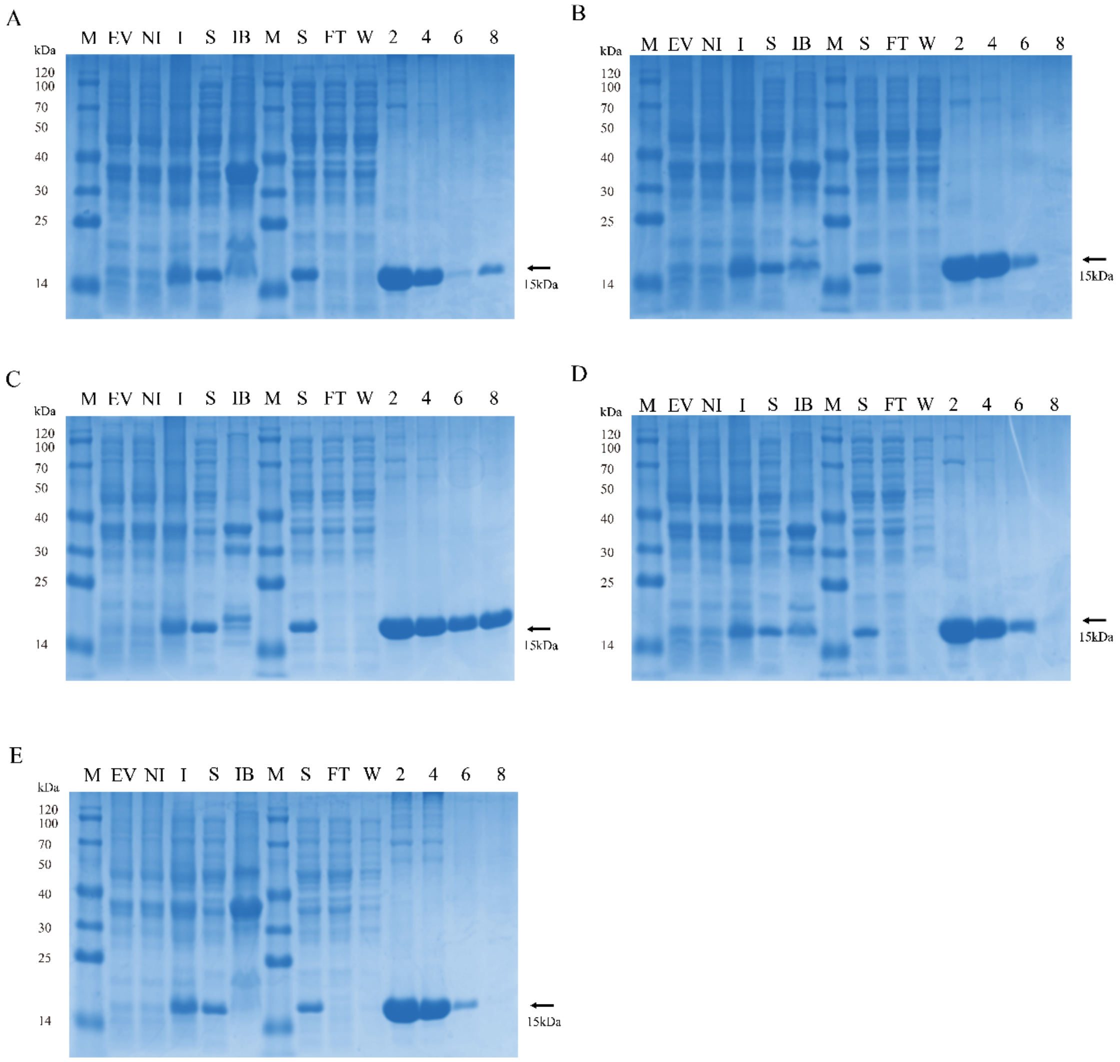

Fig. S2 Expression and purification of soluble nanobodies. (A) Expression and purification of Nb6. Lane M: Protein marker; Lane EV: pComb3Xss vector control; Lane NI: Before Nb6 induction; Lane I: After Nb6 induction; Lane S: Supernatant from cell lysate; Lane IB: Precipitate from cell lysate; Lane FT: Filtrate; Lane W: Wash buffer; Lanes 2–8: Nanoantibodies collected in different EP tubes. (A) Expression and purification of Nb25. (A) Expression and purification of Nb31. Lane arrangement order as above. (A) Expression and purification of Nb36. Lane arrangement order as above. (A) Expression and purification of Nb85. Lane arrangement order as above.

Table S1 Hydrogen bonds between GP4 and Nb6

No	Structure 1	Dist	Structure 2
1	A:SER 78[OG]	3.70	B:ASP 110[OD1]
2	A:ARG 141[HH12]	1.85	B:GLU 44[OE1]
3	A:SER 85[OG]	2.12	B:GLN 1[HE21]
4	A:SER 88[OG]	2.45	B:GLN 1[HE22]

Table S2 Salt bridges between GP4 and Nb6

No	Structure 1	Dist	Structure 2
1	A:ARG 141[NH1]	2.77	B:GLU 44[OE1]
2	A:ARG 141[NH1]	3.96	B:GLU 44[OE2]
3	A:ARG 141[NH2]	2.99	B:GLU 44[OE1]
4	A:ARG 141[NH2]	2.97	B:GLU 44[OE2]

Table S3 Hydrogen bonds between GP4 and Nb31

No	Structure 1	Dist	Structure 2
1	A:LYS 117[HZ2]	1.64	B:GLN 1[OE1]
2	A:LYS 2[HZ3]	1.70	B:GLN 3[OE1]
3	A:LYS 117[HZ1]	1.84	B:GLU 26[OE1]
4	A:LYS 117[HZ3]	1.78	B:GLU 26[OE2]
5	A:SER 79[OG]	3.90	B:TYR 96[OH]
6	A:LYS 2[HZ1]	1.76	B:TRP 103[O]
7	A:LYS 2[HZ2]	1.72	B:GLY 104[O]
8	A:TYR 83[HH]	1.83	B:GLN 105[OE1]
9	A:THR 120[OG1]	2.27	B:ASN 32[HD22]
10	A:GLU 86[OE1]	2.18	B:ARG 45[HH12]
11	A:GLU 86[OE2]	1.76	B:ARG 45[HH11]
12	A:TYR 83[OH]	1.97	B:GLY 106[H]

Table S4 Salt bridges between GP4 and Nb31

No	Structure 1	Dist	Structure 2
1	A:LYS 117[NZ]	2.49	B:GLU 26[OE1]
2	A:LYS 117[NZ]	2.55	B:GLU 26[OE2]
3	A:GLU 86[OE2]	3.98	B:ARG 45[NE]
4	A:GLU 86[OE1]	2.63	B:ARG 45[NH1]
5	A:GLU 86[OE2]	2.72	B:ARG 45[NH1]

Table S5 Hydrogen bonds between GP4 and Nb85

No	Structure 1	Dist	Structure 2
1	A:SER 78[OG]	3.19	B:LYS 107[O]
2	A:SER 78[O]	2.48	B:ARG 100[HH12]
3	A:SER 79[OG]	1.64	B:LYS 107[HZ2]
4	A:SER 85[OG]	1.92	B:GLN 1[HE21]
5	A:ALA 143[O]	2.41	B:ASN 110[HD21]
