# Supplementary figures and images for: Nanobodies Targeting the GP4 Protein Inhibit PRRSV Replication
Source: Microorganisms. 2025 Nov 2;13(11):2524. doi: 10.3390/microorganisms13112524 (PMC12654830; doi:10.3390/microorganisms13112524)

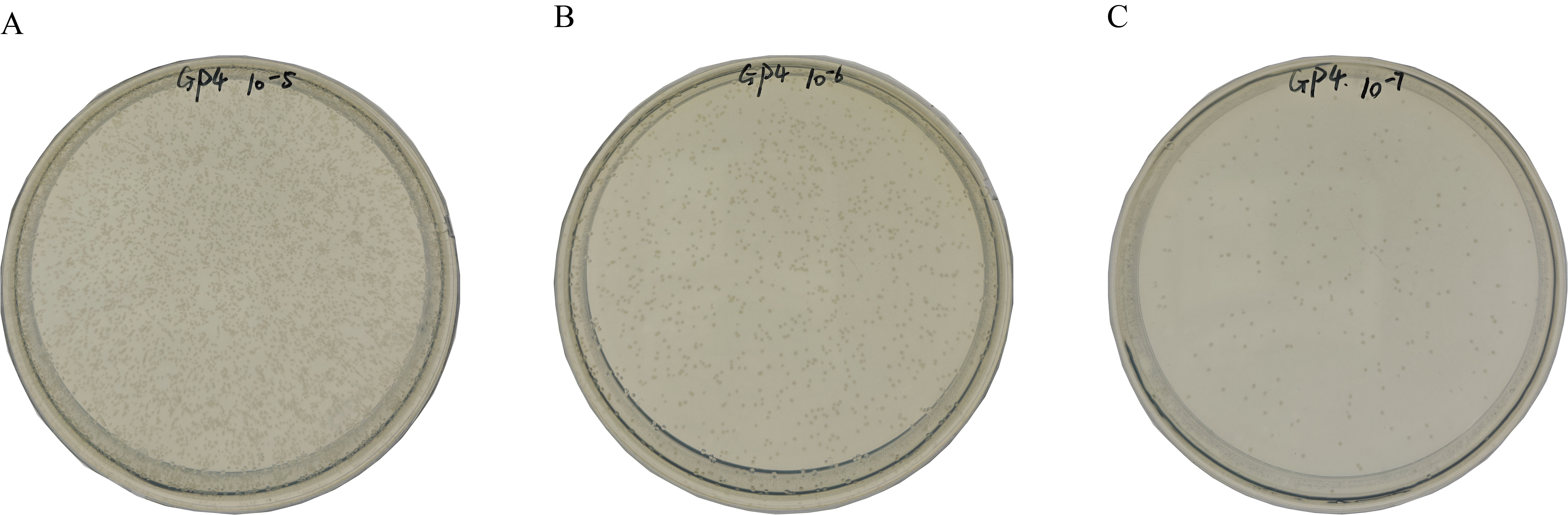

Supplement: Supplementary file 1 [file microorganisms-13-02524-s001.zip › Fig S1.png]
